# *Poria cocos* polysaccharides improve alcoholic liver disease by interfering with ferroptosis through NRF2 regulation

**DOI:** 10.18632/aging.205693

**Published:** 2024-03-20

**Authors:** Xiangyu Zhou, Jincheng Wang, Sufang Zhou

**Affiliations:** 1 Guizhou University of Traditional Chinese Medicine, Guiyang 550002, China; 2 The First Affiliated Hospital of Guizhou University of Traditional Chinese Medicine, Guiyang 550001, China

**Keywords:** inflammatory factors, alcoholic liver injury, NF-Kβ, oxidative stress

## Abstract

The active ingredient in *Poria cocos*, a parasitic plant belonging to the family Polyporaceae, is *Poria cocos* polysaccharide (PCP). PCP exhibits liver protection and anti-inflammatory effects, although its effect on alcoholic liver disease (ALD) remains unstudied. This study investigated the mechanism of PCP in improving ALD by regulating the Nrf2 signaling pathway. After daily intragastric administration of high-grade liquor for 4 hours, each drug group received PCPs or the ferroptosis inhibitor ferrostatin-1. The Nrf2 inhibitor ML385 (100 mg/kg/day) group was intraperitoneally injected, after which PCP (100 mg/kg/day) was administered by gavage. Samples were collected after 6 weeks for liver function and blood lipid analysis using an automatic biochemical analyzer. In the alcoholic liver injury cell model established with 150 mM alcohol, the drug group was pretreated with PCP, Fer-1, and ML385, and subsequent results were analyzed. The results revealed that PCP intervention significantly reduced liver function and blood lipid levels in alcohol-fed rats, along with decreased lipid deposition. PCP notably enhanced Nrf2 signaling expression, regulated oxidative stress levels, inhibited NF-κβ, and its downstream inflammatory signaling pathways. Furthermore, PCP upregulated FTH1 protein expression and reduced intracellular Fe2+, suggesting an improvement in ferroptosis. *In vitro* studies yielded similar results, indicating that PCP can reduce intracellular ferroptosis by regulating oxidative stress and improve alcoholic liver injury by inhibiting the production of inflammatory factors.

## INTRODUCTION

Alcoholic liver disease (ALD) arises from prolonged alcohol consumption, which damages liver cells and gives rise to various related liver diseases. If left untreated, the condition progresses, ultimately leading to alcohol-related hepatocellular carcinoma [1]. Global deaths due to liver cirrhosis and chronic liver diseases have exceeded several million, with approximately 27% attributed to alcohol [2]. Additionally, 245,000 deaths result from alcohol-related liver cancer, constituting 30% of liver cancer fatalities. ALD incidence is on the rise globally (4.5% in China, 6.2% in the United States, and 6.0% in Europe) [3]. As global alcohol consumption continues to increase, the younger demographic engaging in alcohol consumption contributes to a growing population of younger patients with ALD, posing a substantial burden on global public health.

Alcohol abuse is a significant risk factor for ALD, both inducing and exacerbating the disease process. Other risk factors include viral hepatitis, metabolic diseases, overweight, obesity, and smoking [4]. The pathogenesis of ALD is intricate, involving ethanol metabolism, oxidative stress, steatosis, inflammation, and cell death [5, 6]. Current treatment methods include withdrawal therapy, nutritional support, glucocorticoids, and liver transplantation [7]. However, the efficacy of glucocorticoid therapy depends largely on the patient’s conditions and disease stage. Liver transplantation, while a final resort, presents economic challenges and faces a scarcity of liver sources, making it unsuitable as routine treatment. Hence, there is a significant value in exploring new therapeutic targets and researching and developing novel drugs for ALD.

Prolonged alcohol intake intensifies the induction of CYP2E1 [8], an enzyme with a high affinity for nicotinamide adenine dinucleotide phosphate (NADPH) oxidase activity. This leads to the generation of numerous free radicals, causing oxidative stress in the liver. The microsomal ethanol oxidation, dependent on CYP2E1 expression, produces reactive oxygen species (ROS), leading to lipid peroxidation. This process results in the formation of lipid peroxidation products such as 4-hydroxynonenal (4-HNE) and malondialdehyde (MDA), among others [9, 10].

Ferroptosis, a recently identified form of programmed cell death, occurs due to the accumulation of iron-dependent cellular ROS, disrupting cell redox homeostasis [11–13]. Prolonged alcohol intake leads to iron overload in the liver, and iron is known to participate in the Fenton pathway. Accumulated iron ions catalyze hydroxyl radical–mediated oxidative damage in chronic liver disease [14]. This, combined with oxidative stress, exacerbates liver damage [15].

Nuclear factor erythroid 2-related factor 2 (Nrf2) is a crucial regulator of oxidative stress, modulating cell metabolism and mitigating intracellular toxicity [16]. Nrf2 influences genes related to NADPH production, such as malic enzyme 1, glucose 6-phosphate dehydrogenase, isocitrate dehydrogenase 1, and glucose 6-phosphate dehydrogenase, to regulate oxidative stress. Additionally, it induces the reductive activation of key enzymes related to the synthesis and consumption of reduced glutathione (GSH), effectively modulating physiological antioxidant enzymes such as heme oxygenase-1 (HO-1), biliverdin reductase (BVR), and GADPH. Therefore, Nrf2 holds promise as a potential therapeutic target for ALD.

*Poria cocos* polysaccharide (PCP) is the main effective active ingredient in traditional Chinese medicine. *Poria cocos* is primarily composed of glucose, fucose, arabinose, xylose, mannose, and galactose [17]. PCP can functionally intervene in inflammation and confer protection to the liver [18–21]. However, there is a lack of reports regarding interventions with PCPs in alcoholic liver injury. Based on the function of PCPs, this study hypothesized that these polysaccharides can ameliorate alcoholic liver injury by regulating the Nrf2 signaling pathway and interfering with ferroptosis. This study aimed to assess the therapeutic effect on ALD by modulating oxidative stress and improving the ferroptosis pathway in liver cells.

## MATERIALS AND METHODS

### Reagents

Red Star Wine (Beijing Red Star Company Limited, Lot No.: 2021113008); PCP (manufacturer: Solarbio, Lot No.: S98930, UV≥90%); ML385 (manufacturer: APExBIO, Lot No.: BB3003133EF46); ferrostatin-1 (Fer-1; manufacturer: APExBIO, Lot No.: A437141337769); γ-glutamyl transferase (GGT) kit (Cat. No.: C017-2-1), aspartate aminotransferase (AST) kit (Cat. No.: C072-a), alanine aminotransferase (ALT) kit (Cat. No.: C073-a), triglyceride (TG) kit (Cat. No.: C019-a), total cholesterol (T-CHO) kit (Cat. No.: C048-a). All reagents were procured from Changchun Huili Biotechnology Co., Ltd; MDA kit (Cat. No.: A003-1-1), superoxide dismutase (SOD) Kit (WST-1 method; Cat. No.: A001-3-1), GSH kit (microplate method; Cat. No.: A006-2-1), tissue iron kit (colorimetric method; Cat. No.: A039-2-1), and endotoxin kit (limulus reagent method; Cat. No.: E039-1-1). All reagents were purchased from Nanjing Jiancheng Bioengineering Research Co., Ltd. The antibodies Nrf2, HO-1, FTH1, P65, P-P65, and GSH were all procured from Affinity Biosciences (Cat No.: AF0639, AF5393, DF6278, AF2006, AF5006, and DF6214). GPX4 (manufacturer: ABclonal, Cat. No.: A13309) GAPDH (manufacturer: Hangzhou Xianzhi Biological Co., Ltd, Cat. No.: AB-P-R 001), interleukin (IL)-1β, MyD88 (manufacturer: ZEN BIO, Lot No.: L19JL01, LL0414), IL-6 (manufacturer: Bioss Antibodies, Lot No.: BB03079307), protein marker (20-120 KD; manufacturer: GenScript, Cat. No.: M00521). Horseradish peroxidase (HRP)–labeled goat anti-mouse secondary antibody and HRP-labeled goat anti-rabbit secondary antibody were both purchased from Wuhan Boster Biological Engineering Co., Ltd. (Cat. No.: BA1051, BA1054); TRIzoL (manufacturer: Ambion, Lot No.: 15596-026); HiScript® II Q RT SuperMix for quantitative polymerase chain reaction (PCR; +gDNA wiper), rat tumor necrosis factor-α (TNF-α) enzyme-linked immunosorbent assay (ELISA) kit, rat IL-1β (IL-1β) ELISA Kit (manufacturer: Kelu Biotechnology Co., Ltd, Cat. No.: ELK1396, ELK1396, and ELK1272); phosphatase inhibitor (manufacturer: Meilunbio, Cat. No.: MB12707); radioimmunoprecipitation assay (RIPA) lysate buffer (phenylmethylsulfonyl fluoride [PMSF], 100 mM, 1.5 mL; manufacturer: Meilunbio, Cat. No.: MA0151); cytoplasmic and nuclear protein extraction kit (manufacturer: Nanjing KGI Biotech, Cat. No.: KGP150); and ECL substrate solution (manufacturer: Affinity; Cat. No.: KF8003).

### Cell experiment

**Figure 1:**
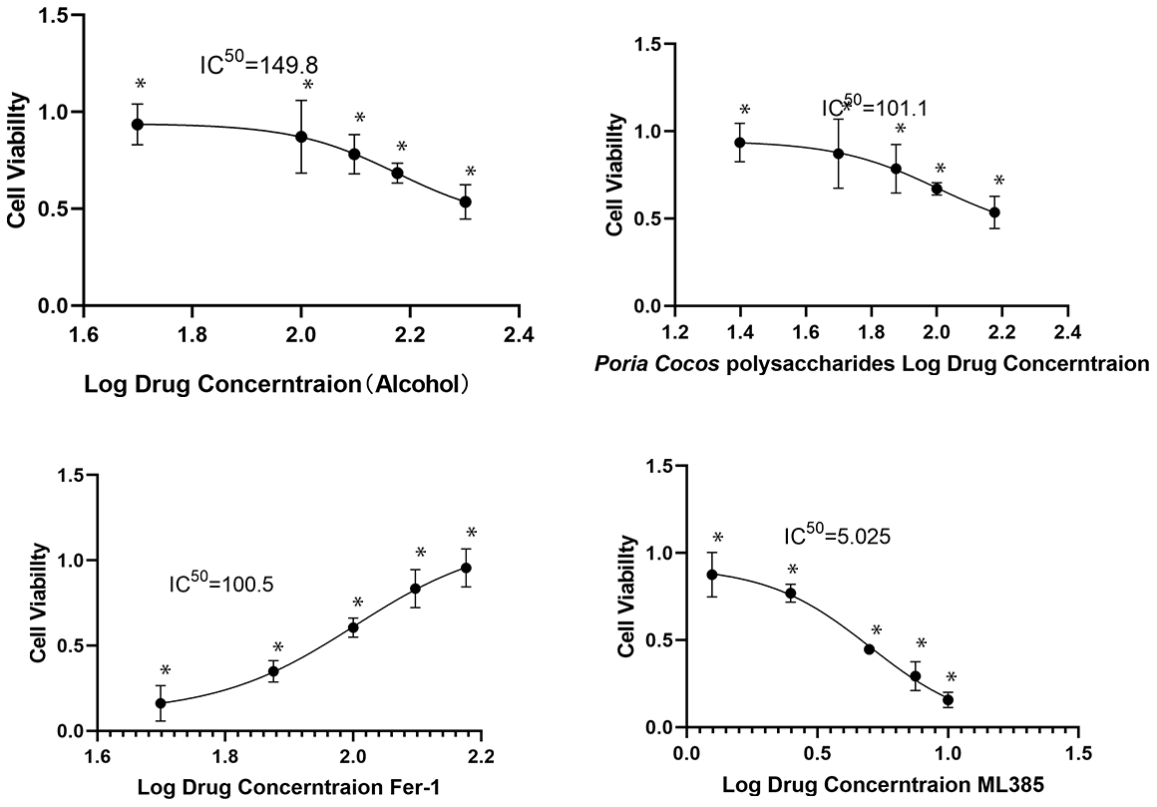
**Different doses of alcohol, *Poria cocos* polysaccharides (PCP), ML385, and Fer-1 were tested on BLR3A cells for 24 hours to determine their effects on cell viability.** The results showed that the optimal intervention concentrations were 150 mM alcohol, 100 μg/mL PCP, 5 μg/mL ML385, and 100 μg/mL Fer-1 (^*^P<0.05).

BRL3A rat liver cells were procured from Wuhan Procell Life Science and Technology Co., Ltd. and maintained in a complete culture medium containing 10% fetal bovine serum, 50 units/mL penicillin, and 50 mg/mL streptomycin. Logarithmically growing cells were employed for experiments and treated with the optimal drug concentration determined by IC_50_ measurement based on CCK8 results (Figure 1). In certain experiments, the cells were pretreated with ML385 (5 μg/mL) for 2 hours before the addition of PCP (100 μg/mL) for 24 hours. Fer-1 (100 μg/mL) served as a drug control group to assess the improvement of PCP on ferroptosis. After 24 hours of drug intervention, the cells were harvested for PCR and protein detection using PCR and western blot analysis.

### Animals

A total of 48 specific pathogen-free (SPF)–grade male Sprague Dawley rats, 8-weeks-old, with a body weight of 220±20 g, were provided by Hunan Experimental Changsha Tianqin Biotechnology Co., Ltd, bearing the experimental animal license number: SCXK (Xiang) 2019-0014. They were raised in separate cages in an SPF-grade experimental environment with a room temperature of 22±2°C and 12 hours of light (with alternating day and night), with free access to food and water. To establish an ALD rat model, all rats were randomly assigned to a control group, ALD group, ALD+ML385 group, ALD+ML385+PCP group, ALD+PCP group, or ALD+Fer-1 group. Rats in the blank group received distilled water of equal volume, while the other groups received high-alcohol white wine (10 mg/kg) [22] by gavage to establish the model. Rats modeled with high-alcohol white wine underwent drug intervention in the second week of gavage: ALD+ML385 (5 μg/kg/day, continuously for 6 weeks), ALD+ML385 (5 μg/kg/day, continuously for 6 weeks)+PCP group (100 mg/kg/day, continuously for 6 weeks), ALD+PCP group (100 mg/kg/day, continuously for 6 weeks), and ALD+Fer-1 group (100 μg/kg/day, continuously for 6 weeks). Rats in the control group and the ALD group received distilled water of equal volume by gavage.

### Determination of liver function, blood lipids, oxidation products, iron ions, inflammatory factors, and GSH

Rat serum was collected, and commercial kits were utilized to measure the levels of AST, ALT, GGT, low-density lipoprotein cholesterol (LDL-C), high-density lipoprotein cholesterol (HDL-C), TC, TG, MDA, SOD, GSH, Fe^2+^, IL-1β, IL-6, TNF-α, and lipopolysaccharide (LPS).

### Oil Red O staining

Liver tissues from rats were obtained, fixed in a 4% paraformaldehyde solution for 48 hours, and subsequently subjected to frozen tissue sections stained with Oil Red O. Pathological alterations in the liver were then examined under a microscope.

### Immunofluorescence method to detect the expression of inflammatory factors, Nrf2, and Fer protein in each group

Cells from each experimental group were trypsinized and plated in a 6-well plate. After fixing the cells on a cell slide, permeabilization was performed with 0.5% Triton X-100 (a detergent prepared with phosphate-buffered saline) at room temperature for 20 minutes. The primary antibody for the target protein, according to the proportions in Table 1, was added to the slide and incubated overnight at 4°C. Subsequently, the corresponding fluorescently labeled secondary antibody (1:400 dilution) was added and incubated at 37°C in the dark for 1 hour. After washing thrice with phosphate-buffered saline containing Tween 20, the cells were stained with DAPI in the dark for 5 minutes, and the slides were mounted with resin glue following treatment with an anti-fluorescence quenching agent. Cell protein staining was observed under a fluorescence microscope. Three random fields of view (×400) were randomly selected from each section, and ImageJ was used to measure fluorescence intensity. Protein expression per unit area was quantified by optical density.

**Table 1:** Antibodies used in immunofluorescence analysis.

**Name**	**Dilution**	**Name**	**Dilution**
Nrf2	1:50	MyD88	1:50
HO-1	1:50	IL-1β	1:50
NF-Kβ	1:50	Fer	1:50

### Western blot

Total protein was extracted from liver tissues and cells using RIPA lysate containing 1% PMSF. The extracted total protein content was measured using a bicinchoninic acid protein assay kit. The proteins in the sample were isolated and transferred onto a polyvinylidene difluoride (PDVF) membrane, which was then blocked with 5% skimmed milk for 4 hours. Following three washes with Tris-buffered saline with Tween 20, the PDVF membrane was incubated with the corresponding primary antibody solution (Table 2) overnight at 4°C on a shaker. Subsequently, the PDVF membrane was washed and incubated with the corresponding secondary antibody solution at room temperature for 2 hours. The ECL reagent was used for the reaction, and the PDVF membrane was developed in a developing device. The gray value of the film was analyzed using ImageJ.

**Table 2:** Antibodies used in Western blotting.

**Name**	**Dilution**	**Name**	**Dilution**
GAPDH	1:5000	MyD88	1:1000
Nrf2	1:1000	GSH	1:1000
HO-1	1:1000	FTH1	1:1000
GPX4	1:1000	GPX4	1:1000
FTH1	1:1000		
P65	1:1000		
P-P65	1:1000		
GPX4	1:1000		

### Quantitative real-time PCR assay

TRIzol reagent was employed to extract total RNA from collected cells and tissues. OD_260_, OD_280_, and OD_260_/OD_280_ values were measured using a micro-spectrophotometer to assess RNA purity and concentration. Total RNA was stored in a −80°C refrigerator for future use. cRNA was synthesized using a reverse transcription-PCR system, followed by amplification using real-time fluorescent quantitative PCR. Primers were designed based on the gene sequence. Table 3 presents specific primer sequences.

**Table 3:** Primer sequences.

**Gene**	**Primer**	**Sequence (5’-3’)**	**prodTCT lenGTh**
Rat GAPD	Forward	CAAGGCTGAGAATGGGAAGC	127 bp
	Reverse	GAAGACGCCAGTAGACTCCA	
Rat Nrf2	Forward	TGCCCACATTCCCAAACAAG	187bp
	Reverse	GCTATCGAGTGACTGAGCCT	
Rat HO-1	Forward	GAAACAAGCAGAACCCAGTC	225 bp
	Reverse	AGAGGTCACCCAGGTAGCG	
Rat GPX4	Forward	AATTCGCAGCCAAGGACATC	186 bp
	Reverse	GGCCAGGATTCGTAAACCAC	
Rat FTH1	Forward	GGCTGAATGCAATGGAGTGT	186 bp
	Reverse	TCTTGCGTAAGTTGGTCACG	
Rat NF-kB	Forward	GGAGACATCCTTCCGCAAAC	66 bp
	Reverse	AGAGATAGCAGTGGGCCATC	
Rat IL-1b	Forward	GGGATGATGACGACCTGCTA	192 bp
	Reverse	TGTCGTTGCTTGTCTCTCCT	
Rat IL-6	Forward	CCACTGCCTTCCCTACTTCA	186 bp
	Reverse	ACAGTGCATCATCGCTGTTC	
Rat MyD88	Forward	GCATGGTGGTGGTTGTTTCT	96 bp
	Reverse	TCTGTTGGACACCTGGAGAC	
Rat GSH	Forward	AACGTACAGGTGCTGGAAGA	105 bp
	Reverse	AGGATGCATCAGCTCTGTGA	

### Statistical analysis

Statistical analysis of the data was performed using GraphPad Prism 8.0 software. The results are presented as x±s, and group comparisons were conducted through a one-way analysis of variance. Multi-group comparisons were assessed using multiple comparison analysis. A significance level of P<0.05 was considered statistically significant.

### Data availability statement

The datasets used and/or analyzed in the current study are available from the corresponding author upon reasonable request.

## RESULTS

### PCP can improve liver function and blood lipids in ALD rats

**Figure 2:**
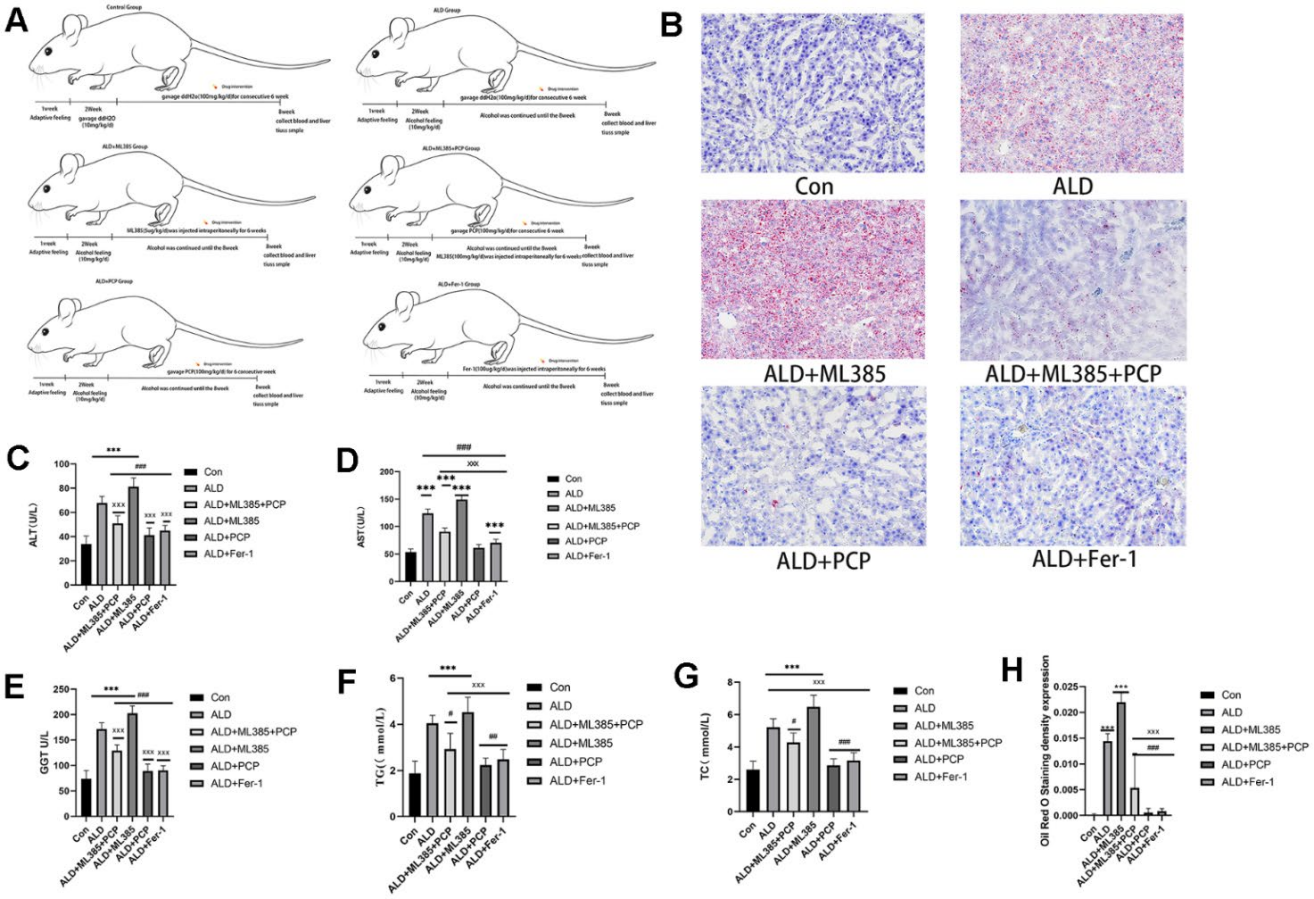
**Comparison of the results of liver function and blood lipid levels in rats in each group.** (**A**) Rat feeding and model building. (**B**) A comparison of the results of hematoxylin and eosin staining and Oil Red O staining in the rat liver. (**C**) A comparison of alanine aminotransaminase results in the rat liver. (**D**) A comparison of aspartate aminotransferase activities in the rat liver. (**E**) A comparison of γ-glutamyl transferase in the rat liver. (**F**) A comparison of triglycerides in the rat liver. (**G**) A comparison of total cholesterol in the rat liver. (**H**) A comparison of the Oil Red O staining results of the liver tissue in rats. The reported values are presented as mean ± SD, n = 8. ^*^P < 0.05, ^***^P < 0.01 compared with the control group; ^#^*P* < 0.05, ^###^*P* < 0.01 compared with the ALD group; ^x^*P* < 0.05, ^xxx^*P* < 0.01 compared with the ML385 group.

Comparatively, the ALD and ALD+ML385 groups exhibited notable hepatic steatosis in rats, evidenced by abundant orange-red lipid droplets, signifying alcohol-induced fat accumulation. By contrast, both the ALD+ML385+PCP and ALD+PCP groups showed a significant reduction in liver fatty degeneration and lipid droplet deposition. The ALD+Fer-1 group demonstrated a reduction in liver fatty degeneration with minimal orange-red lipid droplets. These outcomes are depicted in Figure 2.

To assess the improvement of PCP on rat liver function and blood lipids, serum levels of ALT, AST, GGT, TG, TC, and other indicators were tested. The results showed that the levels of ALT, AST, GGT, TG, and TC in the ALD group and the ALD+ML385 group were significantly higher than those in the control group. Conversely, the ALD+ML385+PCP, ALD+PCP, and ALD+Fer-1 groups exhibited a notable decrease in these indicators. These findings are illustrated in Figure 2.

### Regulation of PCP on oxidative stress and ferroptosis in ALD rats

**Figure 3:**
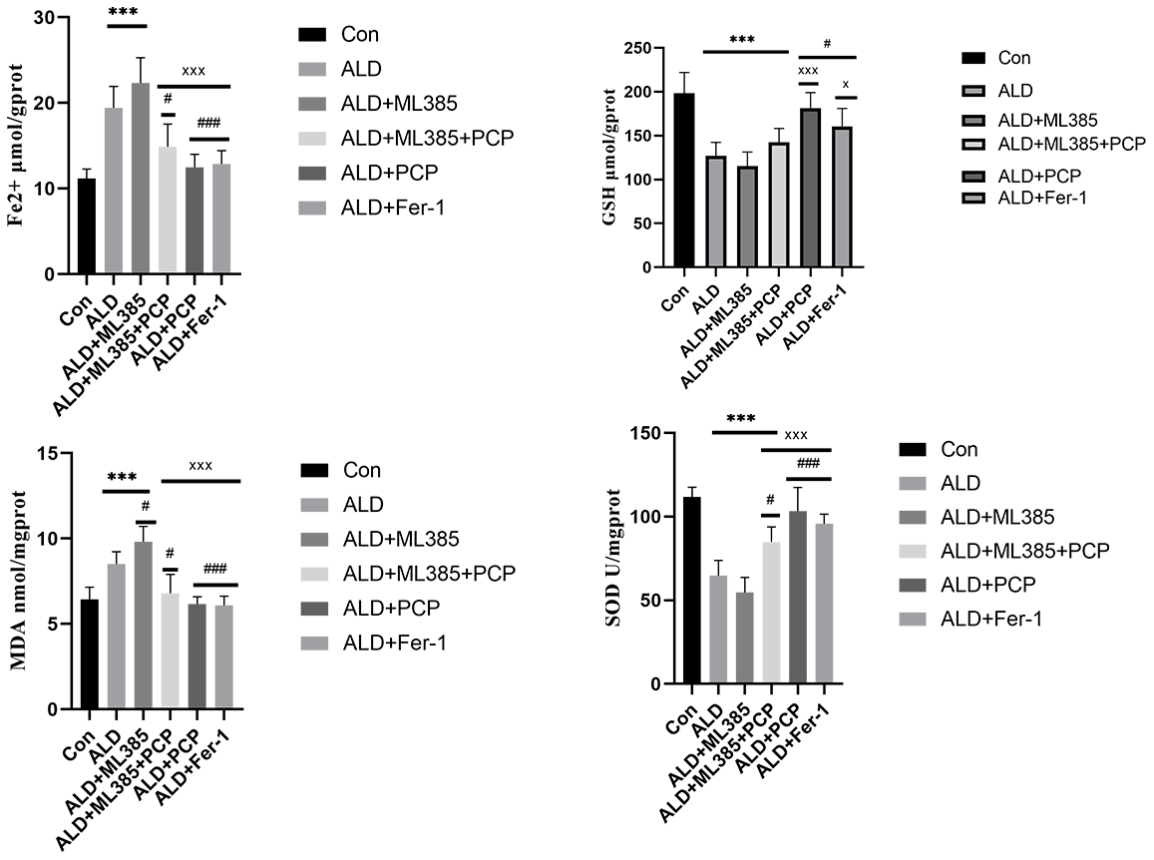
**Effects of *Poria cocos* polysaccharides (PCPs) on oxidative stress and ferroptosis in alcoholic liver disease (ALD) rats.** After the intervention of PCP (100 mg/kg), compared with the ALD group and the ALD+ML385 group, the malondialdehyde (MDA) and Fe^2+^ levels of the rats were significantly reduced (*P*<0.01), and superoxide dismutase (SOD) and glutathione (GSH) were significantly increased (*P*<0.01). After intervention with PCP as well as Fer-1, MDA and Fe^2+^ in rats were significantly reduced (*P*<0.01), and SOD (*P*<0.01) and GSH (*P*<0.05) were significantly increased, indicating that PCP can improve oxidative stress by regulating ferroptosis. The reported values are presented as mean ± SD, n = 8. ^*^P<0.05, ^***^P<0.01, compared with the control group. ^#^*P* < 0.05, ^###^*P* < 0.01, compared with the ALD group; ^x^*P* < 0.05, ^xxx^*P* < 0.01, compared with the ML385 group.

Under the influence of alcohol, a substantial amount of acetaldehyde causes damage to the structure and function of mitochondria. Simultaneously, it generates a significant quantity of ROS, inducing oxidative stress in the body [18]. This oxidative stress state downregulates the expression of hepcidin, leading to iron overload in the liver. The excess iron, in conjunction with oxidative stress, triggers cell death and exacerbates liver injury (Harrison 2006). Compared with the control group, the ALD rat group and the ALD+ML385 group exhibited a marked increase in ROS, lipid oxidation products such as MDA, and Fe^2+^. Additionally, a significant decrease in SOD and GSH was observed. In the treatment group, a substantial decrease in ROS, MDA, and Fe^2+^ was observed, accompanied by a significant increase in SOD and GSH. These findings indicate that alcohol-induced iron deposition in the liver may lead to lipid peroxidation, oxidative stress, and ferroptosis. However, treating this condition proves beneficial in reducing liver iron deposition and minimizing the interaction between iron and oxidative stress. The resultant cell ferroptosis mitigates liver damage. The results are presented in Figure 3.

### Regulation of PCP on serum inflammatory factors in ALD rats

**Figure 4:**
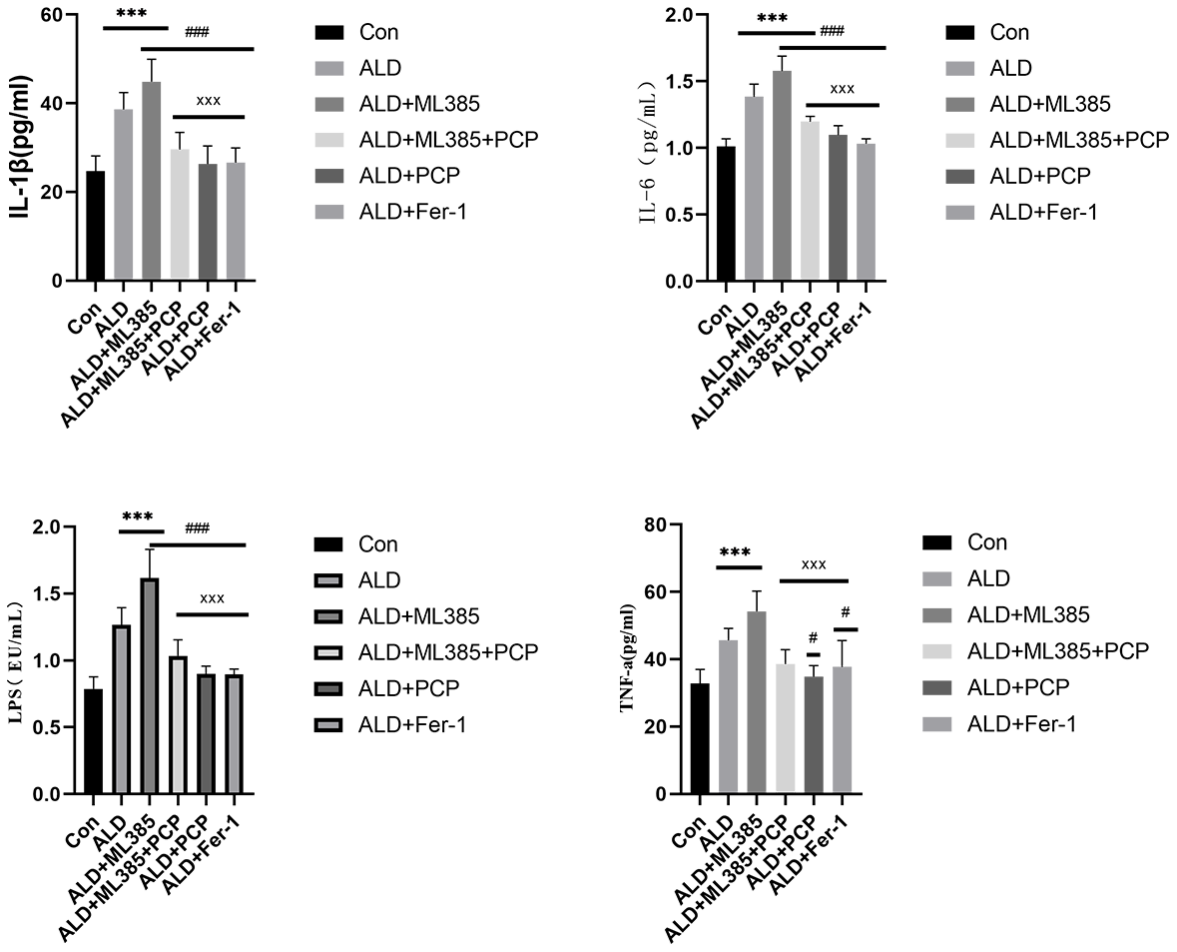
**Effect of *Poria cocos* polysaccharides (PCPs) on inflammatory factors in alcoholic liver disease (ALD) rats.** After the intervention of PCP (100 mg/kg), compared with the ALD group and the ML385 group, the LPS, IL-1β, IL-6, and TNF-α levels in rats were significantly increased (P < 0.01). After intervention with PCP, Fer-1, LPS, IL-1β, IL-6, and TNF-α levels in rats were significantly reduced (*P* < 0.01), indicating that PCP and ferroptosis inhibitors can reduce the expression of inflammatory factors. The reported values are presented as mean ± SD, n = 8. ^*^P < 0.05, ^***^P < 0.01, compared with the control group; ^#^*P* < 0.05, ^###^*P* < 0.01, compared with the ALD group; ^x^*P* < 0.05, ^xxx^*P*< 0.01, compared with the ML385 group.

Prolonged alcohol consumption in patients with ALD results in increased intestinal permeability. Pathogenic factors, such as LPS, enter the hepatic venous circulation through the compromised intestinal barrier. This process activates Kupffer cells and the Toll-like receptor 4 (TLR4)-mediated downstream inflammatory pathway NF-κB, leading to the release of inflammatory cytokines (Wei et al., 2014). Inflammatory factors such as LPS, IL-1β, IL-6, and TNF-α in the ALD group and ML385 were significantly elevated compared with those in the control group. However, in the ALD+PCP group and the ALD+PCP+ML385 group, LPS, IL-1β, IL-6, TNF-α, and other inflammatory factors were significantly reduced. Similarly, the ALD+Fer-1 group exhibited suppressed expression of inflammatory factors. These findings suggest that inhibiting ferroptosis during ALD development can mitigate the production of inflammatory factors and chemokines, thereby ameliorating the effect of inflammation on the liver. The results are presented in Figure 4.

### PCP on the mRNA of inflammatory factors, Nrf2, and ferroptosis in the liver tissue of ALD rats

**Figure 5:**
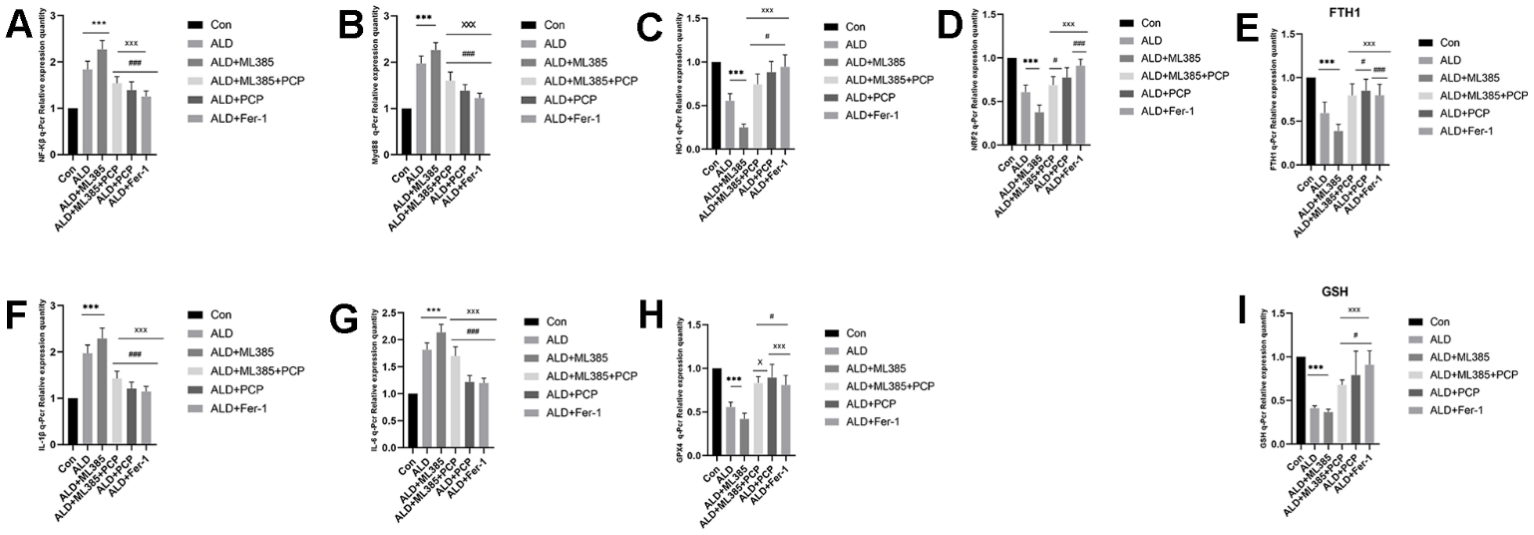
**Effects of *Poria cocos* polysaccharides (PCPs) on the mRNA expression of Nrf2, ferroptosis, and other indicators in the liver tissue of alcoholic liver disease (ALD) rats.** (**A**) NF-κβ. (**B**) A comparison of MyD88 results. (**C**) A comparison of HO-1 results. (**D**) A comparison of Nrf2 results. (**E**) A comparison of FTH1 results. (**F**) A comparison of interleukin (IL)-1β results. (**G**) A comparison of IL-6 results. (**H**) A comparison of GPX4 results. (**I**) A comparison of the results of glutathione. The reported values are presented as mean ± SD, n = 8. ^*^P< 0.05, ^***^P<0.01, compared with the control group; ^#^*P*<0.05, ^###^*P*<0.01, compared with the ALD group; ^x^ *P*<0.05, ^xxx^ *P*<0.01, compared with the ML385 group.

The liver damage caused by alcohol and the impact of PCP were assessed through PCR assays. Compared with the control group, mRNA expression of IL-1β, IL-6, NF-κB, and MyD88 in the liver tissue of rats in the ALD group and the ALD+ML385 group was significantly upregulated. Meanwhile, the expression of Nrf2, FTH1, GSH, GPX4, and HO-1 was significantly reduced. After the intervention of PCP and Fer-1, when compared with rats in the ALD group and the ML385 group, rats in the ALD+ML385+PCP group, ALD+PCP group, and ALD+Fer-1 group showed significantly decreased mRNA expression of IL-1β, IL-6, NF-κβ, and MyD88 in the liver tissue. However, the expression of Nrf2, FTH1, GSH, GPX4, and HO-1 significantly increased. These results are depicted in Figure 5.

### PCP regulation of inflammatory factors, Nrf2, ferroptosis, and other signaling pathways in the liver tissue of ALD rats

To elucidate the specific mechanism by which PCP improves the liver in ALD rats, we investigated signaling pathways related to NF-κB, Nrf2, and ferroptosis. Our findings revealed that, compared with the control group, the expression of P65, P-P65, and MyD88 signals in rats from the ALD group and the ALD+ML385 group was elevated, activating downstream inflammatory factor signaling pathways IL-1β and IL-6. This suggests that after alcohol induction, the NF-κB signaling pathway is activated, leading to an increase in inflammatory response, and iron overload in the liver contributes to ferroptosis. Consequently, the expression of Nrf2, HO-1, FTH1, GSH, and GPX4 signaling pathways decreases.

**Figure 6:**
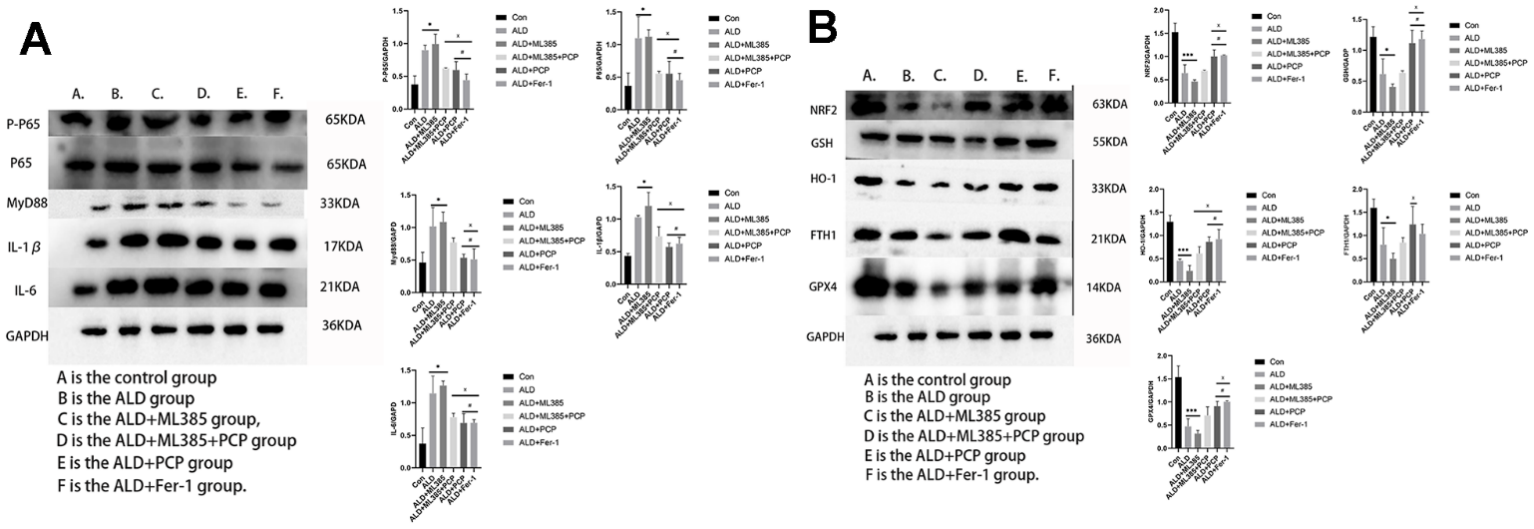
**Effects of *Poria cocos* polysaccharides (PCPs) on the mRNA expression of Nrf2, ferroptosis, and other indicators in the liver tissue of alcoholic liver disease (ALD) rats.** (**A**) The effect of PCP on the inflammatory signaling pathway in the liver tissue of ALD rats. Compared with the control group, interleukin (IL)-1β, IL-6, P65, P-P65, MyD88 were significantly increased in the liver tissue of rats subjected to intervention with alcohol and ML385 (P < 0.05); after the intervention, compared with the ALD model group and the ML385 group, the expression of IL-1β, IL-6, P65, P-P65, and MyD88 in the rat liver tissue was significantly reduced (*P*<0.05). After intervention with PCP and Fer-1, the expression of IL-1β, IL-6, P65, P-P65, and MyD88 in the rat liver tissue was significantly decreased (*P*<0.05). (**B**) The effects of PCP on Nrf2 and ferroptosis-related signaling pathways in the liver tissue of ALD rats. Compared with the control group, Nrf2, GSH, FTH1, HO-1, GPX4 were significantly reduced in the liver tissue of rats after alcohol and ML385 intervention (*P*<0.05); after PCP (100 mg/kg) intervention, compared with the model group and the ALD+ML385 group, the expression of Nrf2, GSH, HO-1, GPX4 in the rat liver tissue was significantly increased (*P*<0.05). After intervention with PCP and Fer-1, the expression of Nrf2, GSH, HO-1, and GPX4 in the liver tissue of rats was significantly increased (*P*<0.05). Although the expression of the FTH1 protein in the ALD+PCP group and Fer-1 showed a downward trend, no significant difference was observed when compared with the ALD group (*P*<0.05). The reported values are presented as mean ± SD, n = 3. ^*^P<0.05, ^***^P<0.01, compared with the control group; ^#^*P*<0.05, ^###^*P*<0.01, compared with the ALD group; ^x^*P*<0.05, ^xxx^*P*<0.01, compared with the ALD+ML385 group.

Additionally, the expression of inflammation and ferroptosis in the ALD+ML385 group was higher than in the ALD group, potentially because of the inhibition of the Nrf2 signaling pathway, and the oxidative stress state aggravates the outcome of inflammation and ferroptosis. However, after drug and inhibitor interventions, the expression of P65, P-P65, and MyD88 signals in the ALD+ML385+PCP group, ALD+PCP group, and ALD+Fer-1 group rats decreased. Simultaneously, the downstream inflammatory factor signaling pathways IL-1β and IL-6 also decreased, and the expression of Nrf2, HO-1, FTH1, GSH, and GPX4 signaling pathways was enhanced. These results are illustrated in Figure 6.

### Effects of alcohol, PCP, Fer-1, and ML385 on the proliferation of BRL3A cells

To mimic alcohol-induced liver injury *in vitro*, BRL3A cells were exposed to alcohol. A cell model was established based on the literature [23, 24]. Initially, we assessed the effect of varying alcohol concentrations (0, 50 mM, 75 mM, 100 mM, 150 mM, and 200 mM) on cell viability at 24 hours. The results indicated that cell survival remained unaffected at an alcohol concentration of 150 mM. Subsequently, an alcoholic liver injury cell model was established using this concentration. We then investigated the effect of different concentrations of PCP (0, 50, 75, 100, 125, and 150 μg/mL), ML385 (0, 1.25, 2.5, 5, 7.5, and 10 μg/mL), and Fer-1 (0, 50, 75, 100, 125, and 150 μg/mL) on cell viability at 24 hours. Experimental results indicated that the optimal intervention concentrations were 100 μg/mL for PCP, 5 μg/mL for ML385, and 100 μg/mL for Fer-1. These results are depicted in Figure 1. Immunofluorescence (IF) detection of inflammatory factors, Nrf2, and Fer protein expression in each group was also performed.

**Figure 7:**
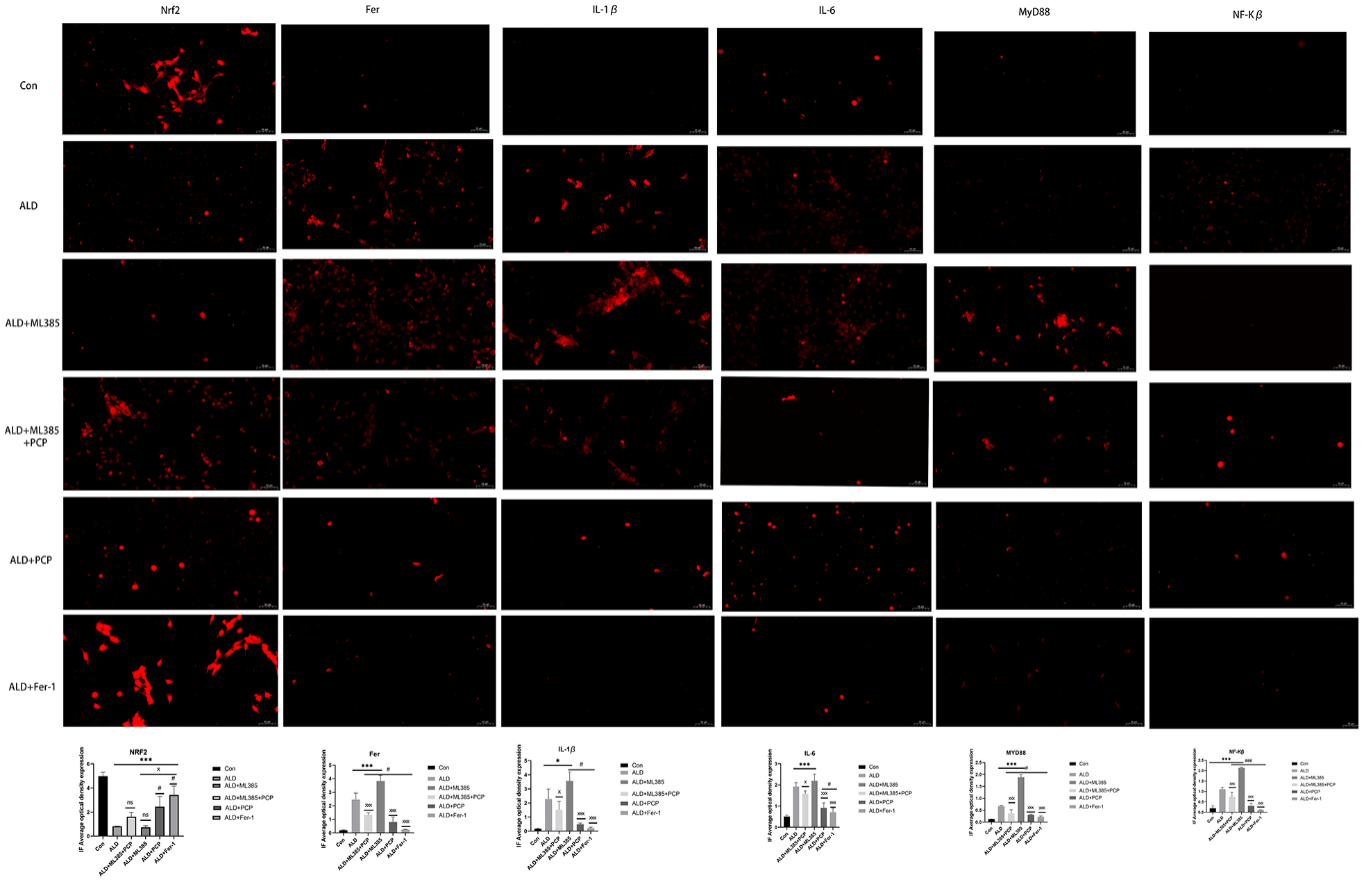
**Effect of *Poria cocos* polysaccharides (PCPs) on intracellular inflammatory factors, Nrf2, and Fer.** After treatment with 150 mM alcohol for 24 hours, the alcohol liver injury cell model was established, and PCP (100 μg/mL) or ferroptosis inhibitor Fer-1 (100 μg/mL) intervention was carried out for 24 hours. After fluorescent staining, inflammatory factors, Nrf2, and Fer expression levels were observed under a fluorescent microscope. The rats were treated with 150 mM alcohol for 24 hours, followed by intervention with the Nrf2 inhibitor ML385 (5 μg/mL) for 2 hours and the addition of PCP (100 μg/mL). The expression of inflammatory factors, Nrf2, and Fer was observed under a fluorescence microscope after fluorescent staining. The reported values are presented as mean ± SD, n =3. ^*^P<0.05, ^***^P<0.01, compared with the control group; ^#^*P*<0.05, ^###^P<0.01, compared with the ALD group; ^x^*P*<0.05, ^xxx^*P*<0.01, compared with the ALD+ML385 group.

The expression levels of inflammatory factors, Nrf2, and Fer in BRL3A cells were assessed through immunofluorescence following alcohol and drug interventions. The results revealed a significant increase in the expression of IL-1β, IL-6, P65, MyD88, and Fe, accompanied by a notable decrease in Nrf2 expression, after alcohol intervention compared with the control group. Similarly, intracellular IL-1β, IL-6, P65, MyD88, and Fe demonstrated a substantial increase, while Nrf2 expression decreased significantly. By contrast, intervention with PCP and Fer-1 showed a significant decrease in the expression of IL-1β, IL-6, P65, MyD88, and Fe, coupled with a significant increase in Nrf2, when compared with the ALD group and the ALD+ML385 group. Figure 7 illustrates these results.

### PCP regulates the mRNA of inflammatory factors, Nrf2, and ferroptosis in liver cells

**Figure 8:**
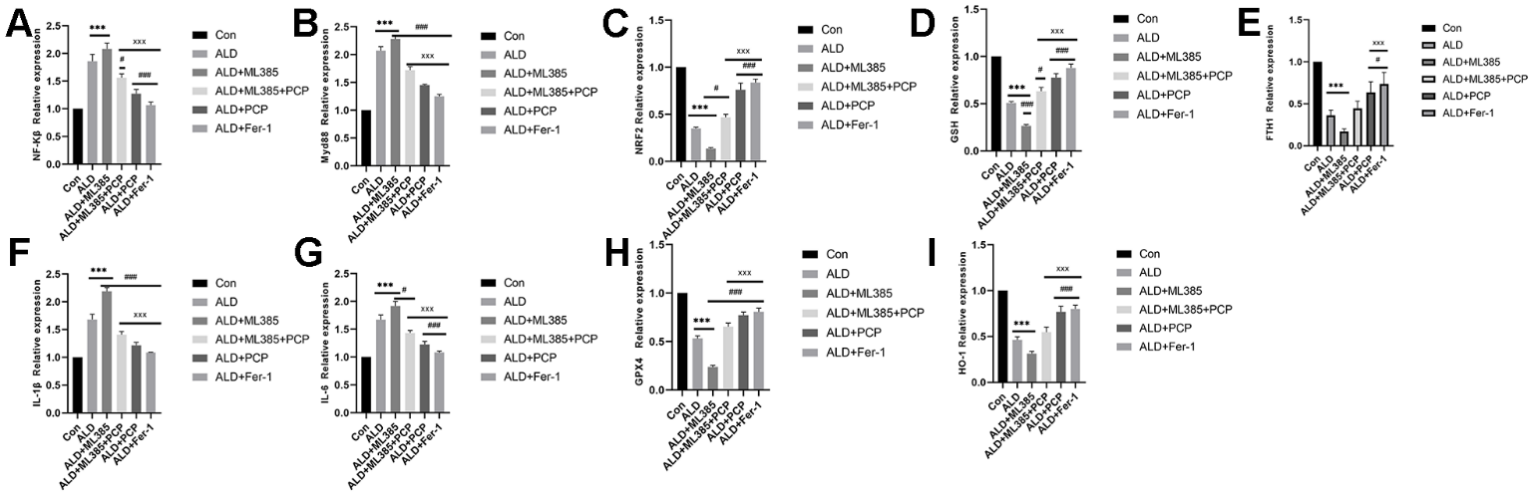
**Effect of *Poria cocos* polysaccharides (PCPs) on the mRNA expression of Nrf2, ferroptosis, and inflammatory factors in BLR3A cells.** (**A**) NF-κβ. (**B**) A comparison of the MyD88 results. (**C**) The comparison of Nrf2 results. (**D**) The comparison of glutathione results. (**E**) A comparison of the FTH1 results. (**F**) A comparison of the interleukin (IL-1β) results. (**G**) A comparison of the IL-6 results. (**H**) A comparison of the GPX4 results. (**I**) A comparison of the results of HO-1. Compared with the control group, after intervention with alcohol and ML385, the mRNA levels of Nrf2, GSH, FTH1, HO-1 and GPX4 in BLR3A cells were significantly reduced (*P*<0.01). After intervention with PCP and Fer-1, compared with the ALD group and the ALD+ML385 group, the mRNA expression of Nrf2, GSH, FTH1, HO-1, and GPX4 in BLR3A cells was significantly increased (*P*<0.05). Compared with the control group, the levels of mRNA of IL-1β, IL-6, NF-κβ and MyD88 after intervention with alcohol and ML385 were significantly increased (*P*<0.01). After intervention with PCP and Fer-1, compared with the ALD group and the ALD+ML385 group, the mRNA expression of IL-1β, IL-6, NF-κβ and MyD88 in BLR3A cells was significantly reduced (P < 0.01). The reported values are presented as mean ± SD, n = 3. ^*^P<0.05, ^***^P<0.01, compared with the control group; ^#^*P*<0.05, ^###^P<0.01, compared with the ALD group; ^x^*P*<0.05, ^xxx^*P*<0.01, compared with the ALD+ML385 group.

In PCR quantification, we observed increased mRNA expression of NF-κβ, IL-1β, IL-6, and MyD88 in BRL3A cells after alcohol and ALD+ML385 treatments. Conversely, Nrf2, HO-1, GPX4, FTH1, GSH, and other mRNA expressions decreased. Treatment with PCP and Fer-1, however, led to increased expression of Nrf2, HO-1, GPX4, FTH1, and GSH, inhibiting ferroptosis and downregulating NF-κβ expression. Assays detecting TNF-α, LPS, IL-1B, IL-6, and other indicators revealed that the ALD+PCP group and the ALD+Fer-1 group had significantly lower levels of TNF-α, LPS, IL-1β, IL-6, and other inflammatory factors. Factor levels were significantly lower in the ALD group and the Nrf2 inhibitor group. The results are depicted in Figure 8.

### PCP upregulates the expression of Nrf2 in liver cells, regulates oxidative stress and ferroptosis, and improves alcohol-induced liver injury

**Figure 9:**
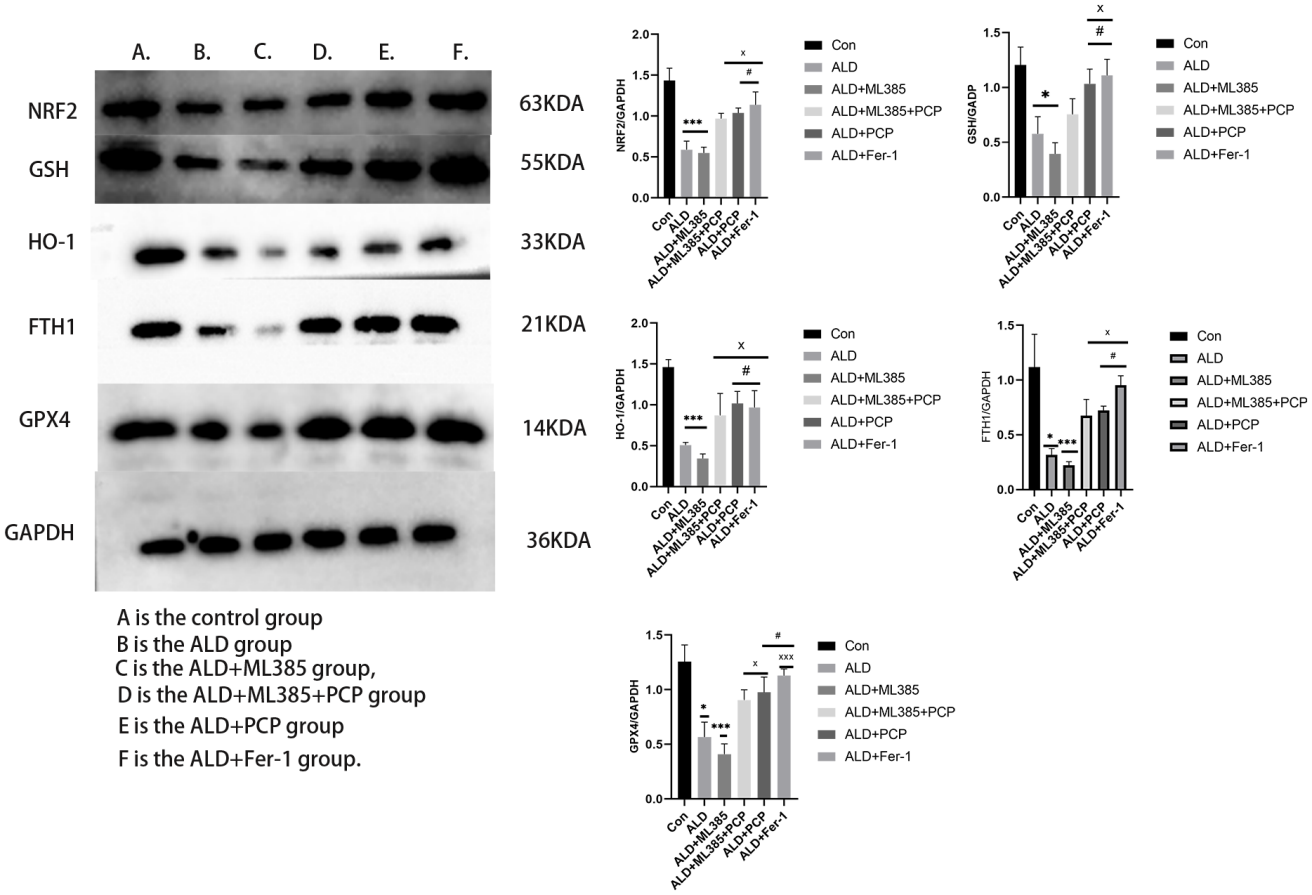
**Effects of *Poria cocos* polysaccharides (PCPs) on Nrf2 and ferroptosis-related signaling pathways in BLR3A cells.** Compared with the control group, the levels of Nrf2, GSH, FTH1, HO-1, GPX4 in BLR3A cells were significantly reduced after the alcohol and ML385 intervention (*P*<0.05); after intervention with PCP (100 μg/mL), compared with the ALD group and the ALD+ML385 group, the expression of Nrf2, GSH, FTH1, HO-1, GPX4, and other mRNAs in rat BLR3A cells was significantly increased (*P*<0.05); after Fer-1 (100 μg/mL) intervention, the expression of Nrf2, GSH, FTH1, HO-1, and GPX4 in the rat liver tissue was significantly increased (*P*<0.05). The reported values are presented as mean ± SD, n = 3. ^*^P<0.05, ^***^P<0.01, compared with the control group; ^#^*P*<0.05, ^###^*P*<0.01, compared with the ALD group; ^x^ *P*<0.05, ^xxx^ *P*<0.01, compared with the ALD+ML385 group.

Alcohol metabolism in the body generates a substantial amount of ROS, leading to oxidative stress, disruption of redox and oxidative defense, inactivation of GSH and GXP4, resulting in lipid peroxidation products, or an excessive iron-induced Fenton reaction. This cascade induces ferroptosis in cells, exacerbating liver damage [25, 26]. In addition to its important role in antioxidative stress, Nrf2 plays a pivotal role in the liver’s response to toxic substances and iron homeostasis [27]. To elucidate PCP’s defense mechanism against alcohol-induced ferroptosis in BRL3A cells, we assessed the expression of the Nrf2 signaling pathway and ferroptosis-related proteins in BRL3A cells. Compared with the ALD group, the expression of Nrf2, FTH1, GPX4, HO-1, and GSH was significantly upregulated in BLR3A cells pretreated with ALD+PCP. A similar result was observed in the ALD+Fer-1 group. In the ALD+ML385 group, however, the expression of Nrf2, FTH1, GPX4, HO-1, and GSH was severely inhibited. The results are shown in Figure 9.

### PCP inhibits the NF-κβ signaling pathway in liver cells and improves alcoholic liver injury

**Figure 10:**
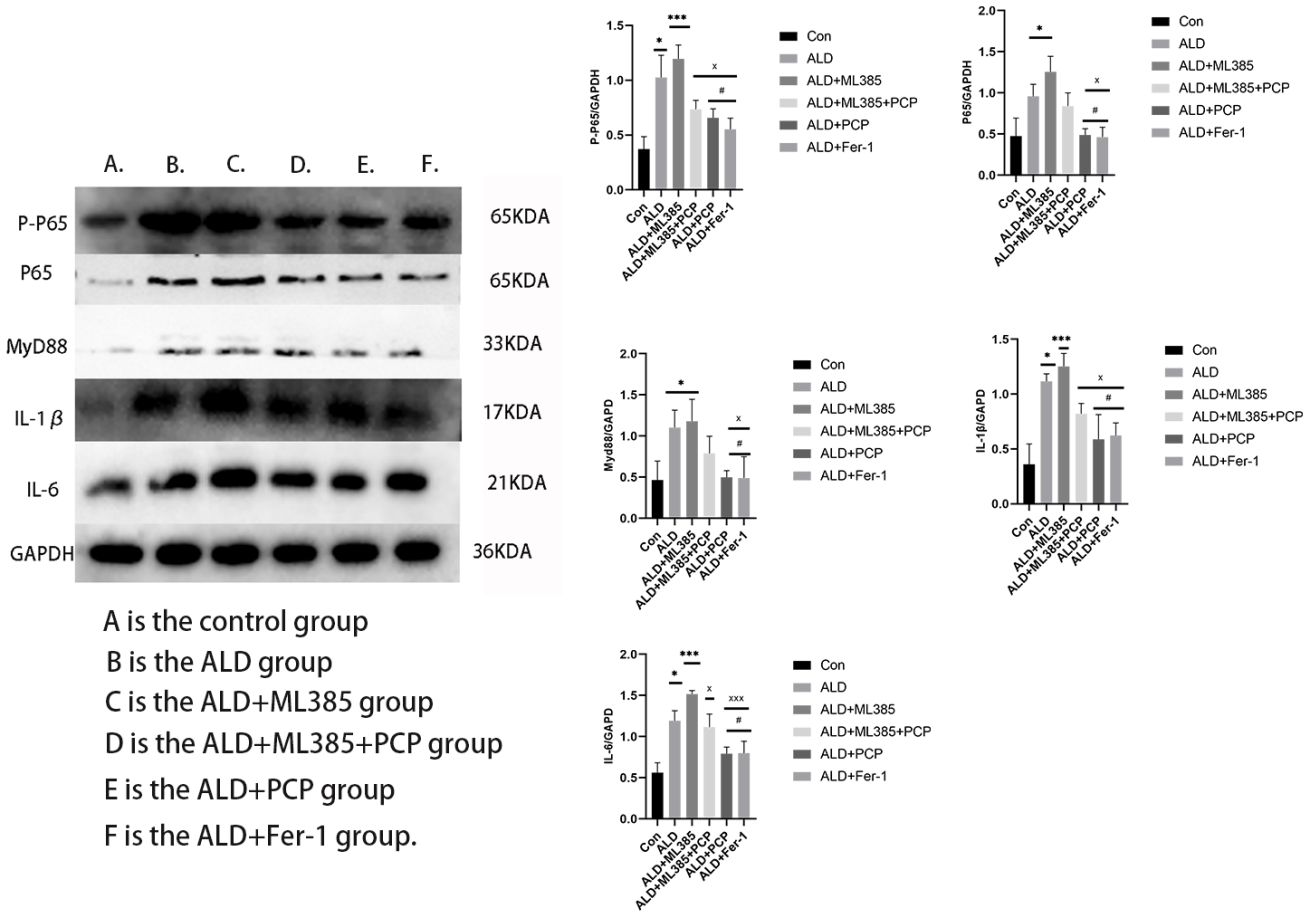
**Effect of *Poria cocos* polysaccharides (PCPs) on the inflammatory signaling pathway of BLR3A cells.** Compared with the control group, levels of interleukin (IL)-1β, IL-6, P65, P-P65, MyD88 in BLR3A cells significantly increased after alcohol and ML385 intervention (P < 0.05); compared with the ALD group and the ALD+ML385 group, after PCP intervention (100 μg/mL), Nrf2, IL-1β, IL-6, P65, P-P65, MyD88 in BLR3A cells were significantly reduced (P < 0.05); after Fer-1 (100 μg/mL) intervention, the expression of IL-1β, IL-6, P65, P-P65, and MyD88 in BLR3A cells was significantly decreased (P < 0.05). The reported values are presented as mean ± SD, n = 3. ^*^P<0.05, ^***^P<0.01, compared with the control group; ^#^*P*<0.05, ^###^*P*<0.01, compared with the ALD group; ^x^*P*<0.05, ^xxx^*P*<0.01, compared with the ALD+ML385 group. Where A is the control group, B is the ALD group, C is the ALD+ML385 group, D is the ALD+ML385+PCP group, E is the ALD+PCP group, and F is the ALD+Fer-1 group.

Under oxidative stress, Nrf2 inhibition enhances the activation of NF-κB, thereby mediating the production of inflammatory factors [28] and accelerating ALD development. Consequently, this study investigated the NF-κB-mediated expression of inflammatory cytokine signals in ALD and BRL3A cells in the ALD+ML385 group. The findings demonstrated that compared with the control group, P-P65 was activated in both the ALD and ALD+ML385 treatment groups. Upregulated expression of P65, enhanced MyD88 signal expression, and increased IL-1β and IL-6 were observed. Notably, the ALD+ML385 group showed higher expression than the ALD group. Conversely, in BRL3A cells pretreated with PCP, the expression of P-P65, P65, and MyD88 significantly decreased, inhibiting the signals of the downstream inflammatory factors IL-1β and IL-6. Furthermore, in the ALD+Fer-1 group, the expression of related inflammatory factors was inhibited. These results are depicted in Figure 10.

## DISCUSSION

Early intervention in alcoholic steatosis and inflammation is crucial in the development of ALD. Therefore, identifying compounds that can alleviate alcohol-induced hepatic steatosis and inflammation may be beneficial for ALD treatment. Despite reports on PCP’s hepatoprotective, antioxidant, and anti-inflammatory mechanisms, there is no relevant information on its efficacy in improving ALD. Thus, we investigated whether PCP can enhance liver inflammation and steatosis in ALD rats. To explore its specific mechanism and its connection to oxidative stress and ferroptosis, we used Nrf2 inhibitors and ferroptosis inhibitors as a positive control group. *In vitro*, alcohol was used to intervene with BLR3A cells to verify pathway-related expressions. The results showed that pachycosin significantly improved fatty degeneration in ALD rats, reduced TG, TC, LDL-C, and HDL-C levels, and improved liver function markers AST, ALT, and GGT, demonstrating a protective effect on hepatic steatosis and liver function. *In vitro*, pachyman increased Nrf2 expression in BRL3A cells after alcohol injury, improved oxidative stress, reduced cell ferroptosis, and inhibited the NF-κB signaling pathway, resulting in reduced inflammatory factor formation and improved steatosis and liver function. Similar effects were observed in the Fer-1 group. In the Nrf2 inhibitor group, oxidative stress increased significantly, NF-κB expression was enhanced, and pro-inflammatory factors were upregulated [29, 30]. These results indicate that Nrf2 plays a crucial role in ALD development, and PCP can improve ALD by regulating Nrf2.

Nrf2 improves ALD through various mechanisms. Alcohol metabolism generates substantial ROS, inhibiting antioxidant enzyme expression, thereby reducing the body’s antioxidant potential and stimulating oxidative stress [31]. Nrf2, a key player in oxidative stress defense, activates the expression of antioxidant-related genes by binding to antioxidant response elements [32]. The study results demonstrated that compared with the ALD and ML385 groups, PCP and Fer-1 enhanced the Nrf2 signal in BRL3A cells, increased Ho-1 levels, strengthened antioxidant ability, reduced lipid peroxidation product MDA, inhibited Keap-1 activity, decreased ROS generation, and consequently inhibited lipogenesis and accumulation, improving alcohol-induced hepatic steatosis.

Alcohol impedes hepcidin expression, diminishing the body’s iron absorption capability and causing elevated iron levels. Consequently, ALD patients often experience excessive iron levels [33], triggering oxidative stress through the Fenton reaction and generating ROS. In the process, GSH is consumed, GXP4 activity is inhibited, lipid peroxidation occurs, inducing cell ferroptosis, and exacerbating ALD development [34, 35]. Our research on BLR3A cells treated with alcohol and Nrf2 inhibitors demonstrated decreased FTH1 and GXP signal expression, reduced GSH content, and elevated Fe^2+^ levels. This indicates that alcohol induction and inhibition of the Nrf2 signaling pathway leads to significant iron deposition, promoting cell death. Notably, these adverse effects were mitigated in cells treated with PCP and ferroptosis inhibitors.

Long-term alcohol consumption can compromise the integrity of the intestinal barrier, allowing intestinal bacteria and LPS to enter the bloodstream through the hepatic vein. This, in turn, activates Kupffer cells, triggering a substantial release of inflammatory cytokines [17]. Activation of inflammatory factors in the liver induces the production of oxidase, resulting in the generation of ROS, thereby accelerating ALD progression through the interplay of oxidative stress and inflammation [36]. Our observations revealed that, in BLR3A cells treated with alcohol and Nrf2 inhibitors, there was a significant enhancement in the expression of the NF-κβ signal. Simultaneously, the up-regulation of its downstream signaling pathway, MyD88, and increased protein expression of inflammatory factors IL-1β and IL-6 were observed. This suggests that inhibiting Nrf2 leads to an upsurge in inflammation. Numerous studies [29, 30] have demonstrated that IL-1β expedites ALD progression by amplifying the production of pro-inflammatory cytokines, upregulating fatty acid synthesis, inducing liver steatosis, and promoting liver fibrosis. Additionally, in patients with ALD, TNF-α and IL-6 have been implicated in hastening disease progression and concurrent multiorgan failure [37]. In BLR3A cells treated with PCP, NF-κβ was significantly inhibited, leading to a reduction in downstream inflammatory signaling pathways. This same phenomenon was observed in BLR3A cells with Fer-1. This indicates that PCP can inhibit the expression of NF-κβ and its downstream pathway, MyD88, thereby reducing the production of inflammatory factors and ameliorating liver injury. The underlying mechanism may be associated with the activation of the Nrf2 signaling pathway, leading to a decrease in ROS generation and cell ferroptosis.
